# Evaluation of Twenty Genes in Prognosis of Patients with Ovarian Cancer Using Four Different Clustering Methods

**DOI:** 10.31557/APJCP.2021.22.6.1781

**Published:** 2021-06

**Authors:** Saeedeh Pourahmad, Somayeh Foroozani, Mehdi Nourelahi, Ahmad Hosseini, Mahboobeh Razmkhah

**Affiliations:** 1 *Bioinformatics and Computational Biology *R*esearch Center, Shiraz University of Medical Sciences, Shiraz, Iran. *; 2 *Department of Biostatistics, School of Medicine, Shiraz University of Medical Sciences, Shiraz, Iran. *; 3 *Department of computer Science, University of Wyoming, Laramie, WY, USA. *; 4 *Shiraz Institute for Cancer Research, School of Medicine, Shiraz University of Medical Sciences, Shiraz, Iran.*

**Keywords:** Gene expression, ovarian cancer, clustering methods, prognosis

## Abstract

**Background:**

Comparison of gene expression algorithms may be beneficial for obtaining disease pattern or grouping patients based on the gene expression profile. The current study aimed to investigate whether the knowledge within these data is able to group the ovarian cancer patients with similar disease pattern.

**Methods:**

Four different clustering methods were applied on 20 genes expression data of 37 women with ovarian cancer. All selected genes in this study had prominent roles in the control of the activity of the immune system, as well as the chemotaxis, angiogenesis, apoptosis, and etc. Comparison of different clustering methods such as K-means, Hierarchical, Density-Based Spatial Clustering of Applications with Noise (DBSCAN) and Expectation-Maximization (EM) algorithm was the other aim of the present study. In addition, the percentage of correct prediction, Robustness-Performance Trade-off (RPT), and Silhouette criteria were used to evaluate the performance of clustering methods.

**Results:**

Six out of 20 genes (IFN-γ, Foxp3, IL-4, BCL-2, Oct4 and survivin) selected by the Laplacian score showed key roles in the development of ovarian cancer and their prognostic values were clinically and statistically confirmed. The results indicated proper capability of the expression pattern of these genes in grouping the patients with similar prognosis, i.e. patients alive after 5 years or dead (62.12%).

**Conclusion:**

The results revealed the better performance for k-means and hierarchical clustering methods, and confirmed the fact that by using the expression profile of these genes, patients with similar behavior can be grouped in the same cluster with acceptable accuracy level. Certainly, the useful information from these data may contribute to the prediction of prognosis in ovarian cancer patients along with other features of patients.

## Introduction

According to the statistical reports, ovarian cancer leads to the highest level of fatality among the women with malignant tumors and is recognized as the fifth cause of death due to cancer (Pashaei-Asl et al., 2017). Due to the lack of specific features, most of the ovarian cancers are diagnosed when they are at their final stage. Therefore, this type of disease has a high treatment cost as well as low prognosis. Nowadays, by understanding the molecular basis of this disease, a new treatment has been proposed to manage this disease (Pashaei-Asl et al., 2017). In fact, various types of cancer could be diagnosed through the analysis of genetic data. All cells of an organism have similar genes, but these genes have different interpretations at different instances and circumstances (Crick, 1970). Biologists measure the gene expression level in different experimental conditions in order to analyze the genes performance and the mechanism regulating them in cancer patients (Grotkjær et al., 2006). One of the main issues in analysis of the gene expression data is to distinguish the gene groups which show a similar expression pattern (Alavi Majd and Tabatabaei, 2014). Therefore, using the gene expression analysis, genes or samples with similar behavior or function are identified (Hand and Heard, 2005). However, as the genes expression in the body affect each other, it seems essential to use the methods which take into account the simultaneous effects of different genes at different diseases, especially in cancers. Furthermore, due to special complexity in distinguishing a disease generating agents, individual gene examination may not lead to accurate conclusions (Shahraki et al., 2016). Accordingly, utilizing multivariate statistical analysis methods in examination of gene performance in different diseases has received attention by the researchers interested in this field (Shahraki et al., 2016). Among these, clustering methods are the most important instruments to study the gene microarray data (Grotkjær et al., 2006; Alavi Majd and Tabatabaei, 2014; Alavimajd et al., 2007; Vahedi et al., 2008; Farhadi and Shahsavani, 2015; Ciaramella and Staiano, 2019; Nies et al., 2019). Clustering can figure out the structure of biomedical data which is helpful for diagnosis or treatment (Nies et al., 2019). 

The main objective of this research was to cluster the patients using their data expression of 20 genes which, based on the clinical literature, are the most effective genes in prediction, diagnosis and treatment of the ovarian cancer (Windbichler et al., 2000; Sers et al., 2002; Marth et al., 2004; Manrique et al., 2011; Bankaitis and Fingleton, 2015; Berinstein et al., 2015; Shang et al., 2015; Comisso et al., 2017; Razaghi et al., 2017; Yokoyama et al., 2017). Since one cannot exactly determine which method is always the most appropriate clustering method for a data type (Ciaramella and Staiano, 2019), comparison of different methods on a data set is of great importance. Accordingly, comparison of different clustering methods such as K-means, Hierarchical, Density-Based Spatial Clustering of Applications with Noise (DBSCAN) and Expectation-Maximization (EM) algorithm was the other aim of the present study. In order to evaluate the performance of clustering methods, we utilized the patients’ prognosis (based on their survival status 5 years after diagnosis) as a criterion. Therefore, the ability of the gene expression data in grouping the patients with similar prognosis was assessed.

## Methods and Materials

*Materials*

*Study Design and Participants*

A prospective study was performed on 37 women diagnosed with ovarian cancer during the year 2007 to 2010. Patients were recruited from a hospital affiliated with Shiraz University of Medical Sciences (south of Iran) and followed for 5 years. Only newly diagnosed patients were enrolled in this study, and definite diagnosis was made after surgical removal of the suspicious ovarian mass and pathologic examination. The type and grade of tumors were determined by an experienced pathologist. All participants provided written informed consent prior to enrollment in the study. 

*Blood samples, RNA Extraction and Reverse Transcription*

Peripheral blood samples were drawn from ovarian cancer patients who did not receive any radiotherapy, chemotherapy, or immunotherapy prior to sampling. The total RNA of blood cells was extracted by lysis with ammonium chloride and Trizol reagent treatment (Invitrogen, USA). Then, the quality and quantity of RNA samples were measured by spectrometry at 260 and 280 nm. Contaminated DNA was removed from RNA by DNase I treatment (Fermentase, Lithuania) before cDNA synthesis. cDNA was synthesized from 0.5-μg of total RNA with the RevertAid First Strand cDNA Synthesis Kit (Fermentase, Lithuania), using both oligo(dt)18 and random hexamer primers.

*Methods*

*Quantitative Real-Time RT-PCR (qRT-PCR)*

Specific primers were designed to determine the expression of IFN-γ, Foxp3, IL-4, IL-6, IL-10, IL-12, IL-17, IL-23, IL-27, BCL-2, Oct4, survivin, SDF-1, CTLA-4, TGF-β, Fas, FasL, Her2, MDM2, P53 (Table 1), and Beta-actin, using Primer-Blast online software. The quantity and expression of gene transcripts were determined using a Bio-Rad system (Chromo4 Real-time PCR Detector, Bio-Rad, USA) for quantitative Real-Time PCR (qRT-PCR) and SYBR Green PCR Master Mix (Applied Biosystems, USA). Expression of β-actin housekeeping gene was used to normalize the expression level of the target gene. Every PCR reaction was done in a final volume of 20 μL that contained 0.5-μg of the cDNA product, 150nM of each primer, and 1× reaction mixture consisting of FastStart gold DNA polymerase, dNTPs, reaction buffer and SYBR green I (Applied Biosystems). The thermal cycling used for all genes was set for denaturation step at 95°C for 10 min, followed by 40 cycles consisting of denaturation at 95°C for15 s, annealing at 56°C for 20 s, and extension at 60°C for 1 min. The qRT-PCR amplification products were verified by melting curve analysis and 1% agarose gel electrophoresis. Amplification efficiency of PCR reaction for all transcripts was determined by plotting a standard curve. The relative quantities of gene transcripts were calculated by the ΔCt and 2^-ΔCt^ formulas.

*Statistical Analysis*

Clustering is a data mining technique that divides different data in separate clusters. In this method, the observations of heterogeneous population are divided into homogenous subsets named clusters. The purpose is to find the groups much different while their members are matched (Vahedi et al., 2008). Most of the clustering methods are based on the criteria of dissimilarity such as distance function. According to the most famous classification, clustering methods are divided into four categories (Verma et al., 2012), including: 1) Partition-based clustering, 2) Graph-based clustering, 3) Density-based clustering, and 4) Model-based clustering. In the present study, from each category of clustering techniques, a method was applied consisting of K-means, Agglomerative Hierarchical, DBSCAN and EM algorithm, respectively. In the following part, we will briefly introduce the methods used in the present study:

**1)K-means clustering method:** In this method, each observation is assigned to the cluster with the least distance from the average of that cluster (Verma et al., 2012). The number of clusters is determined by the user as an input parameter, and the final result is sensitive to the selection of the initial centers of the clusters. The k points as the primary centers of the clusters are randomly determined and the distance between each data and the center of the clusters is calculated. Each data is then assigned to a cluster that has the shortest distance to the center of that cluster. The center of the new cluster is recalculated by averaging the data contained in each cluster. The distance between each data and the new center of the clusters is recalculated and the data are placed in the new clusters according to the minimum distance. The two previous steps are repeated until the centers of the clusters do not change and convergence is achieved.

**2) Hierarchical clustering method:** This method is introduced as one of the most common methods of data clustering in microarray technology due to the method of displaying and placing individual elements in clusters. Using different proximity criteria for clustering in this algorithm creates different techniques (Tan et al., 2006). The number of clusters is also determined by the user. This method is also sensitive to outliers. Hierarchical clustering can be divided into two main types: Agglomerative (which starts with n clusters and ends with one) and Divisive (which starts with one cluster and ends with n ones), so that the results of both methods are definitive and irreversible. The results of the hierarchical clustering are demonstrative and based on a tree of objects, also known as dendrograms. Also, the Kofentic correlation coefficient is an indicator for evaluating the efficiency and goodness of the hierarchical clustering method; the higher this criterion, the more desirable data clustering.

**3) DBscan clustering method:** Density-based spatial clustering of applications with noise (DBscan) is a density-based, simple, and effective clustering algorithm as it separates high-density areas from low-density areas (Tan et al., 2006). This method was proposed by Ester et al. in 1996 and can be used to identify the clusters of different shapes in a data set, even including noise and outlier data (Ester et al., 1996). This method can find clusters that k-mean method is unable to find. On the other hand, it faces problems such as optimal selection of ε and μ parameters in different conditions and heavy computational load. Two important parameters required for DBscan clustering are epsilon (eps) and minimum points (Minpts); the eps parameter defines the neighborhood radius around point x, and the Minpts parameter specifies the minimum number of neighbors in the “eps” radius. Then, for each main point, if it is not already assigned to a cluster, it creates a new cluster and finds all its connected density points and assigns them to the same cluster as the main cluster. This process is repeated for the remaining points in the data set. Finally, it considers the points that do not belong to a cluster as outlier or noise data.

**4) Clustering method based on EM algorithm: **Expectation-Maximization (EM) algorithm is an unsupervised clustering method which does not need training and is based on complex models. It tries to find parameters of a probability distribution with an iterative approach, so that it maximizes the likelihood function. In general, the input of this algorithm is data set (x), total number of clusters (M), accepted error (e), and maximum number of iterations. For each iteration, it first executes step E (Expectation), in which it obtains the probability of each point belonging to each cluster, and then goes to step M (Maximization), which re-estimates the probability distribution parameter vector of each cluster. This algorithm terminates when the parameters of distribution converge, or a maximum number of iterations is reached (Tan et al., 2006).

For reducing space dimension and removing the extra genes whose information is available in the others, two feature selection methods are utilized leading to three subset of genes: two subsets including the genes with Pearson correlations less than 0.85 in each subset and one subset selected by Laplacian scores (a method based on Graph theory and a distance index) (Kohavi and John, 1997). In addition, the percentage of correct prediction, Robustness-Performance Trade-off (RPT), and Silhouette criteria were applied to evaluate the performance of clustering methods (Vahedi et al., 2008)). Since most of the analyses were based on the Euclidian distance among the gene expression values, data were standardized (centering and scaling) before any analysis.

## Results

Findings were based on information of 20 different genes obtained form 37 women who were suffering from ovarian cancer. Most of the patients were at the first stage of the disease (59.5%) and aged 20 to 30 (27.1%) or 51 to 60 years (24.3%). Twelve patients were unmarried (32.4%) and in most married ones the marriage age was in the age of 16 to 20 years (35.2%). Moreover, 54.1% of them had a favorable prognosis, i.e. they were alive for at least 5 years after diagnosis. Table 1 summarizes the demographic data of the patients. The names of 20 genes with their abbreviations and the mean and standard deviation values of genes expression data derived by the real time PCR method are presented in Table 2. 

Four selected subsets of genes are represented in Table 3. Genes with high Pearson correlation coefficients (0.85 and more) were separated into two distinct subsets (subsets 1 and 2). Laplacian score and hierarchical clustering algorithm were the other two criteria for selecting the genes’ subsets (subsets 3 and 4). 

**Figure 1:**
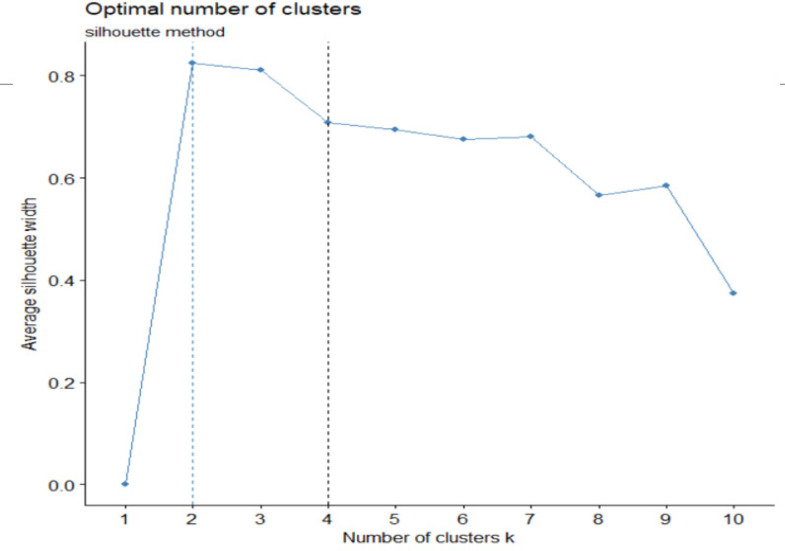
Determining the Number of Optimal Clusters Based on Silhouette Mean Value in k-means Clustering Method

For clustering the patients, four different clustering methods named hierarchical clustering, k-mean, DBSCAN, and EM algorithm were applied on four data sets. Silhouette mean value analysis determined two or four clusters as the optimal number of clusters in different clustering methods. Figure 1 shows the Silhouette mean values for k-means method. For simplicity in interpretation, we represent the results for two clusters. Accordingly, Table 4 summarizes the results of the patients’ clustering into two clusters. The four clustering methods were compared based on Silhouette mean criterion, combined index of RPT, and percentage of correct classification according to the patients’ prognosis (alive 5 years or more / died before 5 years). The results revealed a proper performance of k-means and hierarchical clustering methods. However, their function in gene clustering is clinically debatable.

**Table 1:** Demographic Data for the Available Information of 37 Women with Ovarian Cancer

Variable	The categories	no.(%)	Mean (SD)*
Stage	Stage 1	22 (59.5)	-
	Stage 2	12 (32.4)	
	Stage 3	1 (2.7)	
	Stage 4	2 (5.4)	
	Missing	0 (0)	
Age at diagnosis	Between 20-30	10 (27.1)	25.5 (3.171)
	Between 31-40	6 (16.2)	34.33 (2.944)
	Between 41-50	7 (18.9)	42 (1.414)
	Between 51-60	9 (24.3)	55.11 (3.060)
	Above 61	4 (10.8)	68.5 (9.469)
	Missing	1 (2.7)	-
Age at marriage	Not Married	12 (32.4)	25 (45.227)
	Below 15	3 (8.1)	14.33 (0.577)
	Between 16-20	13 (35.2)	17.69 (1.251)
	Between 21-36	6 (16.2)	29 (5.060)
	Missing	3 (8.1)	-
Prognosis	Favourable (live five years after diagnosis)	20 (54.1)	-
	Unfavourable (dead)	17 (45.9)	-

**Table 2:** Mean Value and Standard Deviation of PCR Real-Time Gene Expression Values for All Cases

Genes’ name (abbreviated)	Full Gene name	Mean (SD)
IL-4	Interleukin-4	0.279 (1.092)
IL-6	Interleukin-6	0.025 (0.089)
IL-10	Interleukin-10	0.012 (0.039)
IL-12b	Interleukin-12	0.064 (0.310)
IL-17	Interleukin-17	0.005 (0.012)
IL-23	Interleukin-23	0.595 (3.040)
IL-27	Interleukin-27	0.006 (0.023)
FoxP3	Forkhead box P3	0.028 (0.129)
CTLA-4	Cytotoxic T-lymphocyte-associated Protein 4	0.010 (0.020)
TGF-β1	Transforming Growth Factor Beta 1	1.761 (3.402)
IFN-γ	Interferon Gamma	0.541 (2.934)
BCL-2	B-cell lymphoma 2	0.899 (3.537)
Fas	-	0.143 (0.606)
FasL	Fas ligand	0.014 (0.056)
Her2	Human Epidermal Growth Factor Receptor 2	0.016 (0.047)
MDM2	Mouse Double Minute 2 Homolog	0.025 (0.055)
Oct4	Octamer Binding Transcription Factor 4	0.215 (0.721)
P53	-	0.306 (1.225)
SDF-1	Stromal Cell-derived Factor 1	0.014 (0.043)
Survivin	-	0.034 (0.128)

**Table 3:** The Selected Subsets of 20 Genes Using Two Feature Selection Methods and the Result of Genes’ Clustering by DBscan Method

The selected genes using pearson correlation coefficient (subset 1)*	The selected genes using pearson correlation coefficient (subset 2)*	The selected genes using Laplacian score	Genes’ clustering using Hierarchical method		
			Cluster 1	Cluster 2	
IL-4	IL-4	IL-4	TGF-β1	IL-4	BCL-2
IL-23	IL-10	FoxP3		IL-6	Fas
IL-27	IL-12b	IFN-γ		IL-10	FasL
CTLA-4	IL-17	BCL-2		IL-12	Her2
TGF-β1	IL-27	Oct4		IL-17	MDM2
Fas	CTLA-4	Survivin		IL-23	Oct4
Her2	TGF-β1			IL-27	P53
MDM2	FasL			FoxP3	SDF1
P53	Her2			CTLA-4	Survivin
Survivin	Oct4			IFN-γ	
	SDF1			IFN-γ	

* The value 0.85 considered for dividing the correlated genes in two different subsets

**Table 4:** The Results of Patients’ Clustering by Four Methods for Two Clusters

Clustering method	Feature selection method	Number of genes in analysis	The Silhoute mean values	RPT criteria	The correct categorization (%)	
					Favourable	Unfavourable
k-means	All genes	20	0.824	1.433	62.16	37.84
	Laplacian score	6	0.881	1.495	56.76	43.24
	pearson correlation coefficient (subset 1)	10	0.721	1.232	54.05	45.95
	pearson correlation coefficient (subset 2)	11	0.784	1.331	54.05	45.95
Hierarchical	All genes	20	0.824	1.433	62.16	37.84
	Laplacian score	6	0.881	1.495	56.76	43.24
	pearson correlation coefficient (subset 1)	10	0.82	1.433	62.16	37.84
	pearson correlation coefficient (subset 2)	11	0.778	1.293	59.46	40.54
DBscan	"All genes ( Ɛ=3 , Minpts=3)"	20	0.703	1.062	54.05	45.95
	"Laplacian score ( Ɛ=1.75 , Minpts=3)"	6	0.809	1.168	54.05	45.95
	"pearson correlation coefficient (subset 1) ( Ɛ=2 , Minpts=3)"	10	0.74	1.162	56.76	43.24
	"pearson correlation coefficient (subset 2) ( Ɛ=2 , Minpts=3)"	11	0.663	1.029	54.05	45.95
EM	All genes	20	-0.131	-0.292	51.35	48.65
	Laplacian score	6	0.324	0.994	54.05	45.95
	pearson correlation coefficient (subset 1)	10	0.338	0.536	48.65	51.35
	pearson correlation coefficient (subset 2)	11	0.712	0.543	48.65	51.35

## Discussion

Since cancer is a complex disease, it is influenced by various factors, such as upregulation of predisposing factors and downregulation of growth-inhibiting factors which ultimately lead to tumor growth and metastasis. Meanwhile, helpful information about genes or disease pattern could be obtained by comparison of gene expression algorithms under different conditions such as diverse tissues, blood specimens and different growing environments (Hand and Heard, 2005; Ciaramella and Staiano, 2019; Nies et al., 2019). All the genes selected in this study had prominent roles in the control of the activity of the immune system, as well as the chemotaxis, angiogenesis, apoptosis, and so forth. Also, the key roles of the genes selected by the Laplacian score including IFN-γ, Foxp3, IL-4, BCL-2, Oct4 and survivin in the development of various cancers and their prognostic value have been clinically confirmed. For instance, Shang et al. reported an association between high FoxP3+ Tregs infiltration and diverse array of solid tumors including cervical, ovarian, renal, and breast cancers (Shang et al., 2015). Another report implies that Foxp3+ regulatory cells are recruited to the tumor microenvironment of high grade serous ovarian cancer and act through immunomodulatory cytokines such as IL-10 and TGF-β (Manrique et al., 2011). Elevated levels of IFN-γ are associated with inducing antitumor immune responses and anti-proliferative activity in the ovarian cancer cells which may mediate through modulating genes involved in cell proliferation or apoptosis (Marth et al., 2004). Furthermore, this cytokine is capable of stimulating the class II tumor suppressor gene H-REV107-1 in the ovarian cancer cells (Sers et al., 2002). Accordingly, treatment of ovarian cancer patients with IFN-γ may improve the survival of these patients (Windbichler et al., 2000; Comisso et al., 2017). Induction of apoptosis can be applied through targeting the genes related to apoptosis such as BCL-2 and survivin which were selected by the Laplacian score in our study. Yokoyama et al. (Yokoyama et al., 2017) suggested a novel combination therapy targeting Bcl-2/Bcl-xL and PARP, leading to greater cytotoxicity against high-grade serous ovarian cancer cells by inducing apoptosis. Also, targeting survivin, as a well-characterized tumor antigen, caused T cell activation, expansion and differentiation in high grade ovarian cancer patients (Berinstein et al., 2015). Other studied genes such as IL-4 and Oct4 which were selected by the Laplacian score are also of great importance in cancer development. For instance, Oct4 can regulate the mitosis and retinoblastoma tumor suppressor pathway. Thus, targeting this pathway may be considered as a potential therapeutic strategy for ovarian cancer (Comisso et al., 2017). Besides, IL-4 and IL-4R pathway induces proliferation, survival and migration of the epithelial cancer cells and thus initiates pro-metastatic related mechanisms which can be prevented by targeting this cytokine axis (Bankaitis and Fingleton, 2015). Collectively, all the genes selected by the Laplacian score in our study were individually effective in the development of ovarian cancer. Thus, we speculate that among the 20 genes studied, these 6 genes may play more important roles in the development and prognosis of ovarian cancer. 

In present study, we made an attempt to investigate whether the knowledge on gene expression data is able to identify the patients with similar disease pattern and categorize them in one group without considering other characteristics of the patients. Among different statistical techniques, clustering methods are of particular interest for this purpose. In gene expression data analysis, clustering can be done for both the genes and patients (Farhadi and Shahsavani, 2015). Therefore, the patients or genes with more similar disease pattern or expression, respectively, are clustered on the same cluster. 

Four clustering methods applied on the present study were the known method of four clustering categories (Shang et al., 2015). The results showed better performance for k-means and hierarchical clustering methods based on Silhouette and RPT criteria (Table 4). In addition, the percentage of correct grouping with similar prognosis was on an acceptable level based on the data of all twenty genes (62.12%). The previous studies confirmed the proper results of these two methods in the diseases diagnosis and patterning based on gene expression data (Grotkjær et al., 2006). According to previous studies, k-means is the most well-known method clustering although it suffers from identifying the number of clusters beforehand (Hand and Heard, 2005; Ciaramella and Staiano, 2019; Nies et al., 2019). This fact may lead to poor performance of k-means in gene expression data clustering (Nies et al., 2019). 

In order to investigate the genes with similar pattern of expression in the present research, hierarchical clustering method was used to cluster the genes. As a result, TGF-β1 was placed in a cluster and the rest in another cluster (Table 3). It is noteworthy that the genes such as IL-4 and FoxP3 which were located in the same cluster prevent the immune system activity, or BCL-2 and Survivin have a role in apoptosis adoption. 

At the end, it should be noted that the better performance for k-means and hierarchical methods may be attributed to lack of noisy data or low sample size (Grotkjær et al., 2006; Verma et al., 2012). In addition, most of the women whose information was used in the current study were on stages 1 or 2 (Table 1), and this fact may lead to the choice of two clusters as the optimal number of clusters. 

Low sample size and unavailability of the other gene expression data can be pointed out as the limitations of the study. Furthermore, simultaneous analysis of this data in both patient and healthy groups and applying other clustering methods (such as soft clustering algorithm) are recommended for future studies.

## Author Contribution Statements

SP was a major contributor to writing the manuscript; she also interpreted the results regarding the statistical methods used. SF and MN were the data analyzer. AH performed data gathering and preparation for the analysis. MR interpreted the patient data and the results of the current study regarding clinical view and also contributed to writing the manuscript. All authors read and approved the final manuscript.

## Data Availability

The datasets analyzed during the current study are available from the corresponding or first author on reasonable request.
